# TiO_2_ treatment using ultrasonication for bubble cavitation generation and efficiency assessment of a dye-sensitized solar cell

**DOI:** 10.1016/j.ultsonch.2022.105933

**Published:** 2022-01-29

**Authors:** Jae-hun Bae, Seong-bin Do, Sung-ho Cho, Kyung-min Lee, Sung-Eun Lee, Tae-Oh Kim

**Affiliations:** a Department of Environmental Engineering, Kumoh National Institute of Technology, Gumi 39253, Republic of Korea; b Department of Applied Biosciences, Kyungpook National University, Daegu 41566, Republic of Korea; c Department of Energy Engineering Convergence, Kumoh National Institute of Technology, Gumi 39177, Republic of Korea

**Keywords:** Ultrasonication, TiO_2_, cavitation bubbles, Surface defects, Dye-sensitized solar cell

## Abstract

In this study, the impacts of different ultrasonic treatments on TiO_2_ particles were determined and they were used to manufacture the photoelectrodes of a dye-sensitized solar cell (DSSC). Two methods were used to prepare TiO_2_ particles directly sonicated by an ultrasonic horn, and TiO_2_ treated indirectly by an ultrasonic cleaner. TEM, XPS analysis was confirmed that cavitation bubbles generated during ultrasonication resulted in defects on the surface of TiO_2_ particles, and the defect induced surface activation. To understand the effect of TiO_2_ surface activation on energy conversion efficiency of DSSC, ultrasonic horn DSSC and ultrasonic cleaner DSSC were prepared. The UV–vis analysis exhibited that the ultrasonic horn DSSC possessed higher dye adsorption when compared to the ultrasonic cleaner DSSC, and the EIS analysis confirmed that the electron mobility was greatly increased in the ultrasonic horn DSSC. The energy conversion efficiency of the ultrasonic horn DSSC was measured to be 3.35%, which is about 45% increase in comparison to that of the non-ultrasonic treated DSSC (2.35%). In addition to this regard, recombination resistance of ultrasonic horn DSSC was calculated to be 450 Ω·cm^2^, increasing more than two times compared to the non-ultrasonic treated DSSC (200 Ω·cm^2^). Taken together, these ultrasonic treatments significantly improved the energy conversion efficiency of DSSC, which was not tried in DSSC-related research, and might lead us to develop more efficient practical route in the manufacturing of DSSC.

## Highlights

• A TiO_2_ photoelectrode is fabricated through a simple ultrasonication treatment.
• Ultrasonic treatment greatly improves particle dispersion and activates TiO_2_ surface.
• Ultrasonic treatment contributes to efficiency improvement.
• A simple and effective sonication is helpful in improving the DSSC efficiency.

## Graphical abstract

**Figure:**
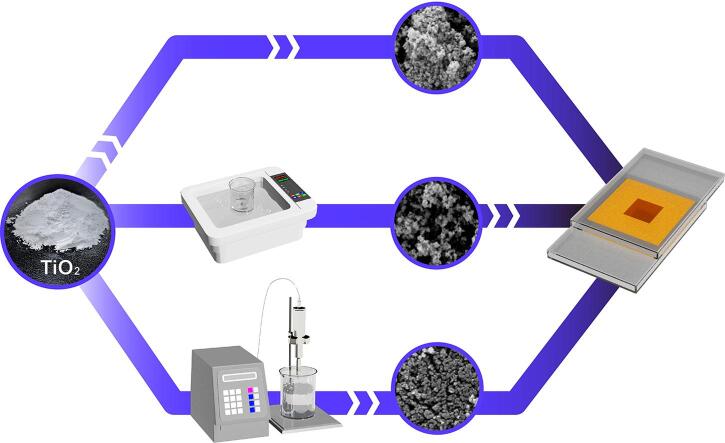

## 1 Introduction

Solar cells are clean and renewable energy sources that do not cause environmental pollution. Depending on their constituent chemicals, solar cells are divided into silicon solar cells [1], [2], thin film solar cells [3], [4], [5], organic solar cell [6], [7], dye-sensitized solar cells (DSSCs) [8], [9], and perovskite solar cells [10], [11]. Among them, silicon solar cells are the most commonly used because they show a high efficiency of 25% or more [1]. However, they are expensive because of their complicated manufacturing process and high energy consumption [2]. To compensate for these disadvantages, many researchers have developed new solar cells to replace silicon solar cells, and one of them is the DSSC.

The DSSC was first introduced by Grätzel in 1991. It generates electricity by irradiating light energy to a dye adsorbed on a semiconductor photoanode, and the excited electrons trigger a redox reaction in the electrolyte [12]. TiO_2_, which is a metal oxide semiconductor, is economical and has high physical and thermal stabilities, making it the most suitable photoanode for DSSCs [8], [12]. Although the DSSC using TiO_2_ has low cost and high stability, it has low dye adsorption and high electron recombination rates, which pose as obstacles to the efficiency improvement [9].

One of the methods of increasing the TiO_2_ photoelectrode efficiency is doping with a transition metal, such as Cu [13], Zn [14], Zr, and W [15]. In our previous studies, doping with various transition metals also improved the TiO_2_ efficiency. Park et al. extended the reactive region of the photoelectrode to the visible region by Cu doping and achieved a high efficiency of up to 11.35% [16]. Meanwhile, Kim et al. improved the dye adsorption by the post-treatment of Zr oxide in TiO_2_ and obtained an efficiency of approximately 7.03% [17]. Lee et al. improved the specific surface area and dye adsorption by doping TiO_2_ with SiO_2_ and N. They enhanced the energy conversion efficiency by up to 8.68% [45]. On the contrary, current studies have encountered difficulties when adding complex heat treatment processes, such as the hydrothermal method, for doping transition metal, which consumes much time to manufacture. Therefore, a new process for improving the TiO_2_ efficiency must be developed.

The methods of preparing TiO_2_ into a photoanode are spin coating [18], dip coating [19], screen printing [20], and doctor blade method [20], among others. Spin coating can smoothly control the voids and thickness of the photoelectrode, but the paste may aggregate at the edges due to a centrifugal force [21]. In dip coating, the photoelectrode thickness is uniform and thin; however, as a limitation, the surface is formed non-uniformly when the paste concentration is high [22]. The screen-printing technology is advantageous for the large-scale production of paste due to uniform thickness. However, in this method, paste is thickly applied, consequently impairing the DSSC efficiency [20]. The doctor blade method is a general photoanode manufacturing method, in which TiO_2_ particles are uniformly distributed inside the paste, and particle loss is small; however, when high-concentration paste is applied, the thickness is not constant, thereby needing improvement [20].

In this study, we will try to improve the energy conversion efficiency of the DSSC by adding a simple and new “sonicated” process to the currently used doctor blade preparation method. Sonication is one of the processes used to form nanoparticles. It leads to surface activation by forming defects on the particle surface as a cavitation reaction [23], [24], [25]. The cavitation reaction is defined as a process in which bubbles in a fluid are formed, grown, and collapsed [26], [27]. It was confirmed that the cavitation reaction is a physical and chemical reaction that oxidizes and activates the catalyst surface [28], [29], [30]. Hafeez et al. reported that the high heat and pressure accompanying the cavitation reaction cause defects and deformation on the particle surface, which contribute to the catalyst activation [23]. Narakaew attempted to improve the activation and reactiveness of the TiO_2_ surface by the cavitation phenomenon [36]. In addition, Stucchi et al. improved the dispersion of the active area by dispersing the particles through ultrasonication [35], [36]. Thus, ultrasonic treatment could results in surface activation and improves the performance of catalysts and photocatalysts by uniformly dispersing materials. However, the sonication process has rarely been applied in the DSSC studies reported so far. Therefore, in the present study, a DSSC was manufactured by applying ultrasonicated TiO_2_ to the photoelectrode. Its applicability and superiority are then investigated. The effect of TiO_2_ treated with ultrasonic cleaner and ultrasonic horn on the DSSC efficiency is also studied.

## 2 Experimental

### 2.1 Materials

The DSSC used in this experiment was prepared using TiO_2_ (anatase 99.9%, US Research Nanomaterials, Houston, TX), α-terpineol (98.5%, Samchun Chemicals, Seoul, South Korea), chloroplatinic acid hydrate (Sigma Aldrich, St. Louis, MO), N719 dye (Solaronix, Aubonne, Swiss), ethanol (HPLC grade, Duksan Co., Ansan, South Korea), and ethyl cellulose 10 cP (extra pure, Daejung Chemicals & Metals, Siheung, South Korea). An ultrasonic cleaner (JAC2010, KODO Technical Research Co., Hwasung-si, South Korea) and an ultrasonic horn (VC750, Sonics & Materials, Newtown, CT) were used as the ultrasonic treatment equipment.

#### 2.1.1 Preparation of the TiO_2_ (commercial TiO_2_) paste

The TiO_2_ paste was prepared through the sol–gel method with 25 ml ethanol, 2.15 ml α-terpinol, and 0.6 ml distilled water added to 2 g of TiO_2_ and stirred at 120 °C at 300 rpm for 15 min. Subsequently, 0.3 g of ethyl cellulose was added and stirred until the paste was completed. The complete paste was applied to the FTO plate by the doctor blade method, which is a method of coating with a constant thickness using a blade, and the process is as follows. In order to prepare a photoelectrode having an area of 5 mm × 5 mm on FTO glass, the other areas were masked. After this process, a certain amount of paste was repeatedly coated on glasses with 5 to 10 times using a blade, and the TiO_2_ photoelectrode was manufactured by a calcination process at 450 °C for 2 h at a temperature increase rate of 5 °C/min. The photoelectrodes were immersed in an N719 dye (0.5 mM) in ethanol at room temperature for 24 h.

#### 2.1.2 Preparation of the ultrasonic cleaner-TiO_2_ paste

The ultrasonic cleaner-TiO_2_ paste manufacturing added an indirect ultrasonic treatment process to the same method for the TiO_2_ paste preparation. After adding 2 g of TiO_2_ to 50 ml of ethanol, ultrasonic treatment (intensity high) was indirectly performed in an ultrasonic cleaner bath for 30 min to prepare an ultrasonic cleaner-TiO_2_ solution. α-Terpinol (2.15 ml) and 0.6 ml distilled water were added to the ultrasonic cleaner-TiO_2_ solution and stirred at 120 °C at 300 rpm for 15 min. After this process, 0.3 g ethyl cellulose was added and stirred until the paste was completed. An ultrasonic cleaner-TiO_2_ photoelectrode was then manufactured in the same manner as that of TiO_2_.

#### 2.1.3 Manufacture of the ultrasonic horn-TiO_2_ paste

The ultrasonic horn-TiO_2_ paste was manufactured in the same manner as the ultrasonic cleaner-TiO_2_ paste. Ultrasonic treatment was directly performed. An ultrasonic horn-TiO_2_ solution was prepared by directly sonicating a solution, in which 2 g of TiO_2_ was added to 50 ml of ethanol with an ultrasonic horn for 30 min. Thereafter, an ultrasonic horn-TiO_2_ photoelectrode was manufactured in the same manner as the ultrasonic cleaner-TiO_2_.

#### 2.1.4 Counter electrode manufacturing and joining

The counter electrode was ultrasonically cleaned in ethanol for 15 min after drilling two holes through which the electrolytes could be injected in the FTO glass. A H_2_PtCl_6_ solution was coated on the washed FTO glass by spin coating and then calcined at 350 °C for 1 h to prepare a counter electrode. After joining the prepared counter electrode and the photoanode, Iodolyte AN-50 was injected as an electrolyte, and the hole was closed to complete the DSSC.

### 2.2 Characterization

The scanning electron microscopy (SEM, ×3000), transmission electron microscopy (TEM, ×250000), Brunauer–Emmett–Teller (BET), and X-ray diffraction (XRD, scan rate 5°/min), and X-ray photoelectron spectroscopy (XPS) analyses were performed in powder form. XRD and XPS were performed to understand the shape and structure of the manufactured TiO_2_. The specific surface area was measured through BET. The surface properties were analyzed through SEM and TEM analyses. The prepared DSSC was analyzed with photo-electrochemical data using 2400 source (Keithley Instruments) under AM 1.5 illumination (100 mW/cm^2^). The electron mobility of the electrode was analyzed by EIS. The chemical capacitance and the recombination resistance were calculated using the EIS and efficiency data.

## 3 Results and discussion

### 3.1 Characteristics analysis of TiO_2_

**Fig. 1:**
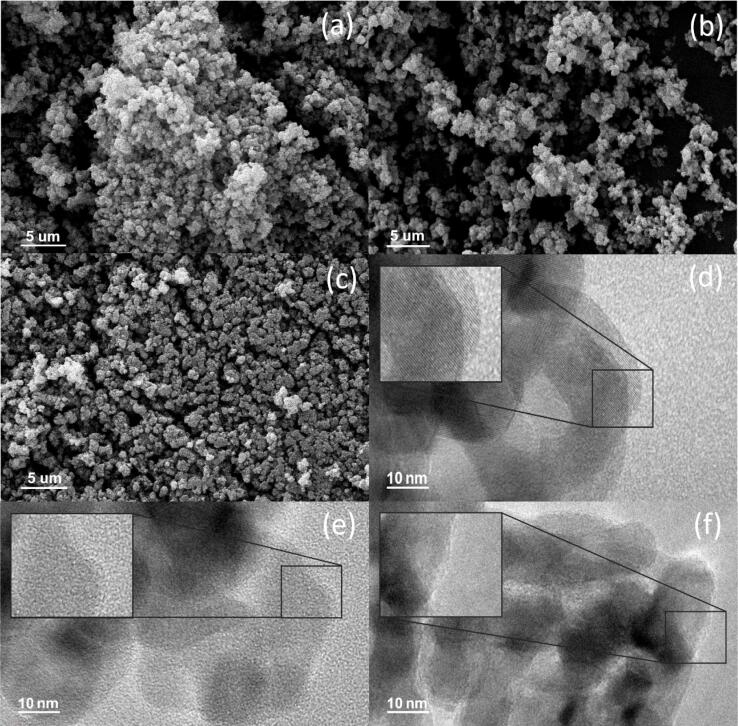
(a-c) SEM images of (a) TiO_2_; (b) ultrasonic cleaner-TiO_2_; (c) ultrasonic horn-TiO_2_. And (d-f) TEM images of (d) TiO_2_; (e) ultrasonic cleaner-TiO_2_; (f) ultrasonic horn-TiO_2_.

The SEM analysis was performed to determine the dispersion of TiO_2_, ultrasonic cleaner-TiO_2_, and ultrasonic horn-TiO_2_ (Fig. 1). The particles in TiO_2_ (Fig. 1 a) were agglomerated, whereas those in the ultrasonically treated ultrasonic cleaner-TiO_2_ and ultrasonic horn-TiO_2_ were dispersed (Fig. 1 b and c). The dispersion degree of the directly applied ultrasonic horn-TiO_2_ was relatively higher than that of the indirectly applied ultrasonic cleaner-TiO_2_, which was consistent with the results of the previous studies showing that ultrasonic waves affect the particle dispersion [36]. The BET analysis was performed to determine the change in the specific surface area before and after the sonication of each TiO_2_ (Table 1). The specific surface areas were measured to be approximately 87.50 m^2^/g for TiO_2_, approximately 88.15 m^2^/g for ultrasonic cleaner-TiO_2_, and approximately 87.44 m^2^/g for ultrasonic horn-TiO_2_. Although the ultrasonic waves affected the particle dispersion, they were thought to not contribute to the changes in the specific surface area.

**Table 1:** Comparison of Brunauer-Emmett-Teller (BET) characteristics of each TiO_2_.

Samples	Textural analysis
	S_BET_ (m^2^/g)
TiO_2_	87.5003
Ultrasonic cleaner-TiO_2_	88.1523
Ultrasonic horn-TiO_2_	87.4388

The previous analysis showed the external change of the TiO_2_ particles by ultrasonication. The changes in the properties of the inside of the particles were confirmed by the results of the TEM, XRD, and XPS analyses. Fig. 1 (e–h) depict the TEM images of each sample measured at 250,000 × magnification. Accordingly, TiO_2_ particles with a diameter ranging from 30 nm to 50 nm were observed. The roughness of the outside of the particle was increased by the ultrasonication, which is more clearly shown in the insets describing TiO_2_ (Fig. 1 e) and ultrasonic horn-TiO_2_ (Fig. 1 f). It was assumed that the ultrasonic wave was applied to the liquid medium (ethanol) mixed with TiO_2_, and the generated cavitation bubble formed a defect on the particle surface. The TiO_2_ surface defects occurred in the form of an oxygen vacancy or Ti^3+^ [31], [32] and did not significantly affect the surface area of the particle itself. It may, however, contribute to the particle surface activation [33]. This is related to the XPS analysis results, which will be described later. The TiO_2_ surface activation caused by the defects improved the photocatalytic performance [34].

**Fig. 2:**
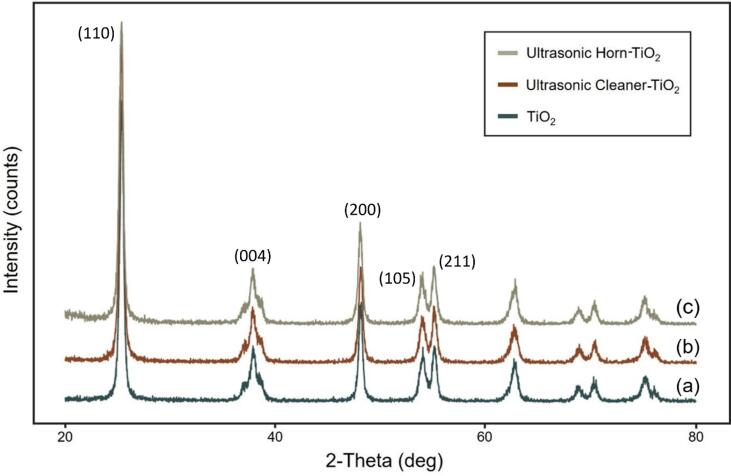
XRD patterns of (a) TiO_2_; (b) ultrasonic cleaner-TiO_2_; (c) ultrasonic horn-TiO_2_.

Fig. 2 shows the XRD analysis results comparing the ultrasonic cleaner/ultrasonic horn-TiO_2_ and TiO_2_. Three spectra with almost identical diffraction peaks were identified, regardless of ultrasonication. The distinct diffraction peaks at 25°, 38°, 48°, 54°, and 55° were consistent with the intrinsic spectrum of the anatase phase TiO_2_ (JCPDS no.: 88–1175 and 84–1286) [37], [38]. This indicates that no change occurred in the crystal compositions of the three TiO_2_ used herein.

**Fig. 3:**
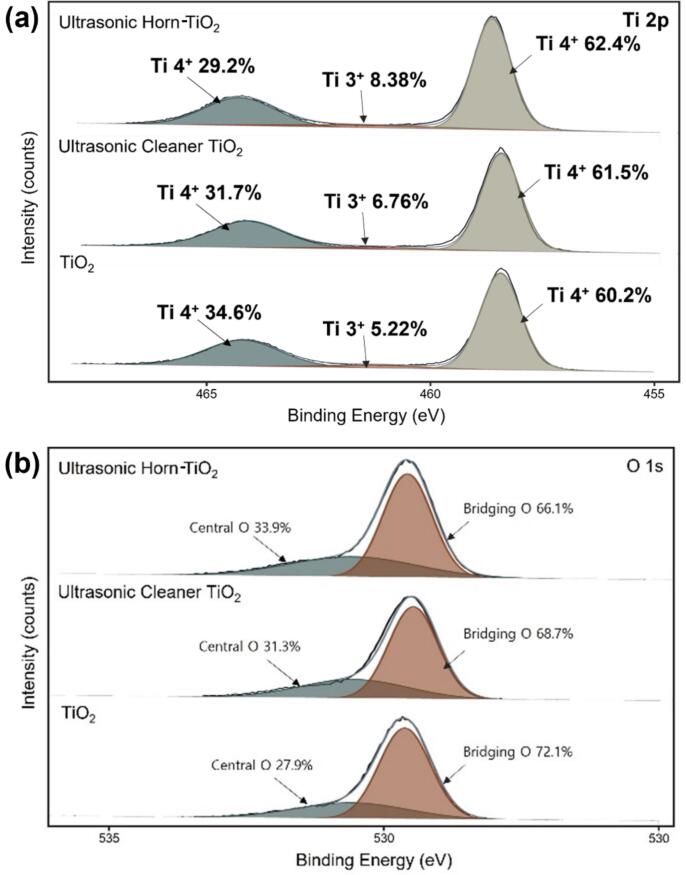
(a) XPS survey spectrum Ti 2p of TiO_2_, ultrasonic cleaner-TiO_2_, ultrasonic horn-TiO_2_; (b) XPS survey spectrum O 1 s of TiO_2_, ultrasonic cleaner-TiO_2_, ultrasonic horn-TiO_2_.

The XPS analysis was performed to analyze the oxidation state of each TiO_2_ surface. Fig. 3 depicts the obtained results. All XPS analyses were corrected for C 1 s (284.6 eV). In the Ti 2p spectrum, two peaks corresponding to Ti^4+^ (i.e., Ti^4+^ 2p_1/2_ and Ti^4+^ 2p_3/2_) and one peak representing Ti^3+^ (i.e., Ti^3+^ 2p_1/2_) were analyzed. The Ti^3+^ 2p_1/2_ peak originated from the oxygen vacancy on the TiO_2_ surface caused by ultrasonication, indicating that the ultrasonic wave activated the TiO_2_ surface [39], [40]. The proportion of Ti^3+^ on each TiO_2_ surface was the highest in the ultrasonic horn-TiO_2_ (8.38%). Those of the ultrasonic cleaner-TiO_2_ and TiO_2_ were 6.76% and 5.22%, respectively. These results demonstrate that ultrasonic treatment induced the TiO_2_ surface activation.

The O 1 s spectrum showed the oxygen species present on the TiO_2_ surface. The peaks corresponding to the lattice oxygen (bridging O, 529–530 eV) and active oxygen (central O, 530–532 eV) of TiO_2_ were found in the O 1 s spectrum [41], [42], [43]. As shown in Fig. 3, the active oxygen in the ultrasonic horn-TiO_2_ was 33.96%; that in the ultrasonic cleaner-TiO_2_ was 31.27%; and that in C-TiO_2_ was 27.89%. These results were consistent with those of the Ti 2p spectrum, in which the ultrasonic treatment improved the active oxygen content of TiO_2_ and activated the TiO_2_ surface.

### 3.2 Characteristics analysis of DSSC

**Fig. 4:**
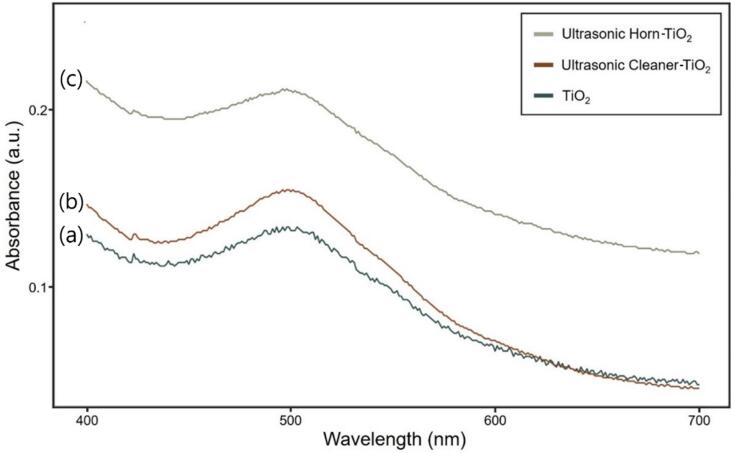
UV–visible absorption spectrum for the dye adsorption assessment. (a) TiO_2_; (b) ultrasonic cleaner-TiO_2_; (c) ultrasonic horn-TiO_2_.

The previous analysis results confirmed that ultrasonic treatment activates the TiO_2_ surface. UV–vis, EIS, J-V graph analyses were employed to prove how the surface activation of TiO_2_ affected the DSSC efficiency. First, the ultraviolet–visible (UV–vis) spectrum was used to check the dye adsorption, which was the main electron source of the DSSC herein, to confirm the increase in the dye adsorption (Fig. 4). To evaluate the amount of dye adsorption, the absorbance was measured by adsorbing dye to the photoelectrode for 24 h, then desorbing it with 1 mM KOH solution [44]. At 500 nm wavelength, the absorbance of the ultrasonic horn was significantly higher than that of the other two samples. The amount of dye adsorbed on the photoelectrode surface was observed in the following order: ultrasonic horn-TiO_2_ > ultrasonic cleaner-TiO_2_ > TiO_2_.

When compared to other two treatments, the ultrasonic horn-TiO_2_ exhibited a higher dye adsorption caused by the particle surface activation, which was due to the cavitation bubbles with particle dispersion [45]. The high dye adsorption of the ultrasonic cleaner-TiO_2_ and the ultrasonic horn-TiO_2_ acted as a key factor for improving the energy conversion efficiency of the DSSC by increasing the current density [46], [47].

**Fig. 5:**
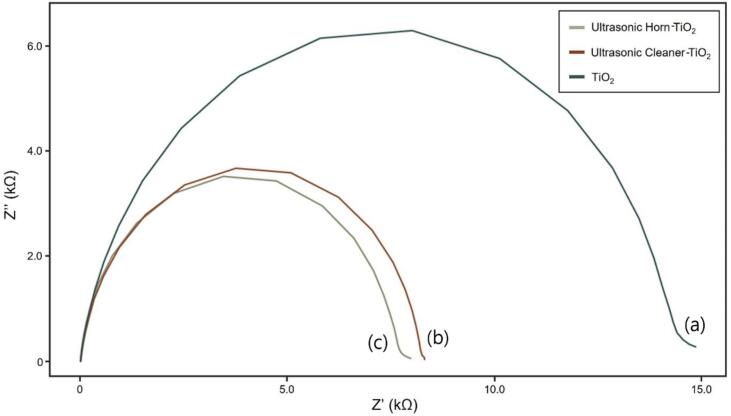
EIS Nyquist plot (under dark condition) of (a) TiO_2_; (b) ultrasonic cleaner-TiO_2_; (c) ultrasonic horn-TiO_2_.

Through the EIS analysis, impedance was measured to determine the electron transfer resistance. Fig. 5 depicts the EIS data observed under dark conditions. The semicircle in Fig. 5 depicts the charge transfer resistance measured at approximately 15 kΩ, with TiO_2_ being the highest. The semicircle size of the ultrasonic cleaner-TiO_2_ and the ultrasonic horn-TiO_2_ was approximately 7–8 kΩ, confirming that the charge transfer resistance was 50% lower than that of TiO_2_. The resistance received by the electrons was low when the charge transfer resistance was low; thus, the current flowed well and may act as a factor for improving the DSSC efficiency.

**Fig. 6:**
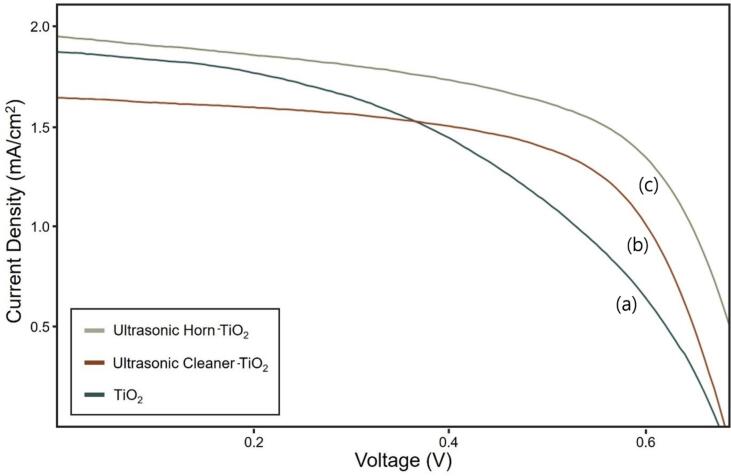
Photocurrent density–voltage (J-V) graph of the DSSCs (under one-sun illumination of 1000 mW/cm^2^). (a) TiO_2_; (b) ultrasonic cleaner-TiO_2_; (c) ultrasonic horn-TiO_2_.

The energy conversion efficiency of each TiO_2_ photoanode was evaluated. Fig. 6 shows the J–V graph measuring the energy conversion efficiency. Table 2 lists the parameters. The energy conversion efficiency of the ultrasonic cleaner-TiO_2_ was 2.82%, which improved by approximately 20% compared to TiO_2_ (2.35%). The ultrasonic horn-TiO_2_ showed an efficiency of 3.35%, which was 43% higher than that of TiO_2_ (2.35%) and approximately 19% higher than that of the ultrasonic cleaner-TiO_2_. The amount of dye adsorption was increased by the dispersion effect of ultrasonication and the activation effect of the TiO_2_ surface, which increased the current density. In conclusion, sonication contributed to the increase of the DSSC efficiency by lowering the electron transfer resistance and increasing the current density.

**Table 2:** Summary of energy conversion efficiency of the DSSCs according to sonication.

Samples	J_sc_ (mA/cm^2^)	V_oc_ (V)	FF (%)	η (%)
TiO_2_	5.54	0.68	45.8	2.35
Ultrasonic cleaner-TiO_2_	6.40	0.68	64.5	2.82
Ultrasonic horn-TiO_2_	7.47	0.72	62.3	3.35

The chemical capacitance (C_μ_) and the recombination resistance (R_r_) were calculated to confirm the electrical characteristics of the DSSCs prepared using TiO_2_ as a photoelectrode. The two calculations were based on the method proposed by Bisquret and the results of our previous study [44], [48], [49], [50]. Table 3 shows the parameters required for the calculation. In the table, ΔE_c_ denotes the difference in E_c_ between each TiO_2_. We found no change in the E_c_ value because the materials were chemically identical to each other. The R_0_ value is a parameter that determines the effect on the recombination process (Eq. (4)). The R_s_ value indicates the series resistance of the DSSC and obtained herein using the EIS data.

(1) $$C_{\mu }=L\left(1-p\right)qg\left(E_{Fn}\right)$$

**Table 3:** Parameters for calculating the chemical capacitance and the recombination resistance. ΔE_c_ means the difference in conduction band energy (E_c_) between each material. Since these samples have the same chemical composition, there is no change in the E_c_ value.

Samples	ΔE_c_	j_0_ (mA/cm^2^)	R_0_ (Ω cm^2^)	R_s_ (kΩ)
TiO_2_	Ref.	5.54 × 10^−3^	3.81 × 10^6^	43.46
Ultrasonic cleaner-TiO_2_	0	6.40 × 10^−3^	4.54 × 10^6^	26.88
Ultrasonic horn-TiO_2_	0	7.50 × 10^−3^	8.30 × 10^6^	25.12

In Eq. (1), *L* denotes the TiO_2_ film thickness; *p* denotes the photoelectrode porosity; and *q* denotes the elementary charge.

(2) $$g\left(E_{Fn}\right)=\frac{\alpha qN_{L}}{k_{B}T}exp\left[\alpha \left(E_{Fn}-E_{C}\right)/k_{B}T\right]$$

Eq. (2) presents the Fermi level (E_Fn_), Boltzmann constant (k_B_), total density of the bandgap states (N_L_), and exponent of the electrons below the conduction band. It is calculated using the constant (α) for the parameter for the exponential trap distribution of electrons under the conduction band.

(3) $$R_{r}=R_{0}exp\left[-\beta \frac{qV_{F}}{k_{B}T}\right]$$

The recombination resistance (*R_r_*) is calculated using Eq. (3). The variable *V_F_* (Fermi level voltage) important for the *R_r_* calculation is calculated using Eq. (5). *V_appl_* represents the applied voltage.

(4) $$R_{0}=\frac{k_{B}T}{\beta qJ_{sc}}exp\left[\frac{\beta qV_{oc}}{k_{B}T}\right]$$

(5) $$V_{appl}=V_{F}-{jR}_{s}$$

(6) $$V_{ecb}=V_{F}-{\Delta E}_{c}/q$$

The equivalent common conduction band voltage (*V_ecb_*) of the *V_F_* value had the same value as the *V_F_* because the material used for each TiO_2_ photoanode is chemically the same. Therefore, the chemical capacitance and the recombination resistance data using *V_ecb_* were not presented herein.

**Fig. 7:**
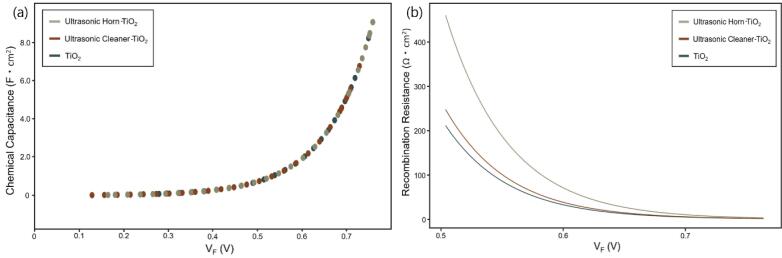
Graphs for the (a) chemical capacitance and the (b) recombination resistance of TiO_2_, ultrasonic cleaner-TiO_2_, ultrasonic horn-TiO_2_.

The chemical capacitance in Fig. 7(a) showed similar C_μ_ values because each DSSC applied to the calculation is chemically the same material. Meanwhile, the recombination resistance in Fig. 7(b) illustrated values of approximately 200 Ω cm^2^ for TiO_2_ and approximately 450 Ω·cm^2^ for ultrasonic horn-TiO_2_ at V_F_ 0.5 V. The R_r_ values increased more than twice because the open-circuit voltage was increased by the effect of the ultrasonic treatment. The increase in the recombination resistance was one of the factors that increased the energy conversion efficiency along with the increases of the dye adsorption and the current density.

## 4 Conclusions

In this study, a TiO_2_ photoelectrode was fabricated through a simple ultrasonication treatment. The surface area change of TiO_2_ was analyzed by performing SEM and TEM analyses. Ultrasonic treatment greatly improved the particle dispersion and activated the TiO_2_ surface. The XRD analysis confirmed that the anatase structure was maintained, even after ultrasonic treatment. The structural TiO_2_ surface activation was analyzed by XPS, and the results showed that the cavitation bubble increased the active area of TiO_2_. The effect of the TiO_2_ surface activation on the DSSC efficiency was investigated through UV–vis and EIS analyses. The UV–vis analysis revealed that sonication significantly increased the dye adsorption. Meanwhile, the EIS analysis confirmed that sonication reduced the charge transfer resistance. Although the chemical capacitance of each TiO_2_ was calculated, the materials used in each DSSC were chemically identical, showing similar capacities. The measurement of the energy conversion efficiency of the DSSC depicted that the DSSC consisting of ultrasonic horn-TiO_2_ had an energy conversion efficiency of 3.35%, displaying approximately 45% of improvement compared to TiO_2_ (2.35%). In addition, the recombination resistance was calculated using the open-circuit voltage and current density values of the DSSC. The results confirmed that ultrasonic treatment contributed to efficiency improvement by increasing the recombination resistance. In conclusion, the simple and effective sonication described in this paper will be very helpful in improving the DSSC efficiency.

## Declaration of Competing Interest

The authors declare that they have no known competing financial interests or personal relationships that could have appeared to influence the work reported in this paper.
